# Adolescents: Contraceptive Knowledge and Use, a Brazilian Study

**DOI:** 10.1100/tsw.2009.8

**Published:** 2009-01-18

**Authors:** Divanise S. Correia, Ana C. P. Pontes, Jairo C. Cavalcante, E. Sócrates T. Egito, Eulália M.C. Maia

**Affiliations:** ^1^ Universidade Federal do Rio Grande do Norte (UFRN), Centro de Ciências da Saúde (CCS), Programa de Pós-graduação em Ciências da Saúde (PPGCSa), 59010-180, Natal-RN, Brazil; ^2^ Universidade Federal de Alagoas (UFAL), Faculdade de Medicina, Campus A C SimÕes Cidade Universitária CEP, 57072-900, Maceió-AL, Brazil

**Keywords:** adolescent, contraceptive methods, pregnancy

## Abstract

The purpose of this study was to identify the knowledge and use of contraceptive methods by female adolescent students. The study was cross-sectional and quantitative, using a semi-structured questionnaire that was administered to 12- to 19-year-old female students in Maceió, Brazil. A representative and randomized sample was calculated, taking into account the number of hospital admissions for curettage. This study was approved by the Human Research Ethics Committee, and Epi Info^TM^ software was used for data and result evaluation using the mean and chi-square statistical test. Our results show that the majority of students know of some contraceptive methods (95.5%), with the barrier/hormonal methods being the most mentioned (72.4%). Abortion and aborting drugs were inaccurately described as contraceptives, and 37.9% of the sexually active girls did not make use of any method. The barrier methods were the most used (35.85%). A significant association was found in the total sample (2,592) between pregnancy and the use of any contraceptive method. This association was not found, however, in the group having an active sexual life (559). The study points to a knowledge of contraceptive methods, especially by teenagers who have already been pregnant, but contraceptives were not adequately used. The low use of chemical methods of contraception brings the risk of pregnancy. Since abortion and aborting drugs were incorrectly cited as contraceptive methods, this implies a nonpreventive attitude towards pregnancy.

## INTRODUCTION

Adolescence is the period of human development characterized by social, psychological, and physical transformations. These transformations are observed and felt by adolescents, and are influenced by their environment[1,2].

The condition of being physically ready to carry out their sexual functions exposes adolescents to the risks of a nonprecautionary sexual life due to their psychological and social immaturity[3]. This shows the need they have not only to be free, but to learn how to deal with their bodies and desires, and to be aware of the objective and subjective repercussions of these attitudes in their lives[4].

Sexuality is one of the aspects that relates to the individual autonomy of youngsters in relation to his/her family, demanding learning on affective and social relationships[3,4,5,6,7,8,9]. In addition, adolescents are starting their sexual lives much earlier everywhere, a situation which renders them vulnerable to the risks of sexually transmitted diseases, to unplanned pregnancies, and abortion[5,6,7,8].

In recent years, the HIV/AIDS outbreak has generated much information that recognizes the importance of sexual behavior and the relationship between this behavior and health[10,11]. Unplanned pregnancy in adolescence, however, has long been a public health problem in Brazil. It is observed that teenagers do know contraceptive methods, but they engage in risky behavior because they do not use them[12,13].

Various factors, such as the degree of information about reproduction and contraceptive actions, questions related to gender, type of affective involvement at the moment, financial issues, and access to contraceptive methods, as well as the level of autonomy reached by teenagers at their age, all influence this behavior[14,15,16].

At the present time, the U.S., for example, is experiencing a decline in pregnancy and abortion cases in the adolescent population. This is due to the use of contraceptives and the fact that more teens are delaying sex[17]. In developing countries, however, the studies point to low utilization of contraceptives by youngsters and a high abortion rate. It is estimated that each year, there are 46 million abortions worldwide and approximately 20 million of these are clandestine[17,18,19,20].

In the adult population, the discrepancy that exists between the number of wanted and unwanted children may be due, in part, to problems caused either by the nonefficacy of contraceptives or by their misuse. However, the use of contraceptive methods in the adolescent population raises different issues in terms of age characteristics[21].

The discussion on the use or nonuse of contraceptive methods and the reproductive and sexual rights achieved by adult women is not the same as for the age group studied here. The nonutilization of any method in adolescence is related much more to questions inherent to their age and immaturity than to the girl's access to these methods[22].

The issue of the knowledge and use of contraceptive methods is highlighted at this age, particularly in countries where girls have an easy access to them and where the number of the so-called unplanned pregnancies is high[6,23,24].

The objective of this study was to identify the knowledge and use of contraceptive methods by a group of female students between the ages of 12 and 19 years. This research is part of a larger study that addresses the reasons for adolescent abortions.

## METHODS AND MATERIALS

### Type of Study and Place

This was a cross-sectional, quantitative study using a semi-structured questionnaire that was administered in ten schools (five public and five private) in Maceió, Alagoas, Brazil.

### Population and Sample

This research is part of a larger study about abortion. Because abortion is an illegal practice in Brazil, there are no official statistics about it. It is therefore necessary to use other statistical methods[25]. In this study, the representative and randomized sample was calculated by taking into consideration the number of hospital admissions for curettage (D&C), according to the suggestion provided by Rossier[25] and the Guttmacher Institute[17].

In Maceió in 2004, the adolescent population between 10 and 19 years of age included 344,221 girls. There were 1,327 hospital admissions for female teenagers for curettage, from a total of 5,622 for all ages. To calculate the sample for this research, and in accordance with the approach of the authors listed above, a figure of 12% of 5,622 was calculated. This value refers indeed to the cases that did not need hospital admission, a total of 675 procedures, from which 25% was deducted. This number refers to 169 spontaneous abortions.

The result of deducting 169 from 675 is 506, a value that was multiplied by a Correction Index of 5, suggested by Correa and Freitas[26], for investigations about abortion in Brazil, which resulted in a minimum sample of 2,530.

This study was undertaken in the Public and Private School System in Maceió and included those schools that have Junior and High School levels, the age group used in the study. The number of students enrolled in each school, in 2004, was searched and there was an estimate that 50% of the students would be female. Then, the number to be found in each school was calculated and it was evident that ten schools would be enough for the attainment of the established sample. From this, the drawing of the educational institutions was carried through, considering 50% for public and 50% for private.

The survey was administered in the random group of schools, and a sample of 2,592 female students between 12 and 19 years of age was obtained. The age selected was from 12 and not 10, the beginning of adolescence identified by the World Health Organization[19]. This was due to the teenagers' interest in responding the questionnaire and the need to obtain authorization from the parents or legal guardians. This represents a sample that provides a confidence interval of 95%.

## Procedures

In the first contact at each school, the objective of the research was explained to the adolescents in the classroom, their concerns were discussed, and the Free and Clear Consent Form was distributed for them to complete along with their parents/guardians. Another time was scheduled for the administration of the questionnaire to those teenagers who wished to participate in the research.

The questionnaire, produced by the researchers after a literature review and tested in a pilot study, was administered in the classroom at a special time, which enabled those female students who wished to participate to do so, after the administrators received the consent forms from both the students and their parents/guardians.

The questionnaires were administered and the data were collected by five groups of two female students from medical and psychological schools, previously trained for this purpose. The choice of those students allowed for the possibility of a dialogue on the theme of "age affinities" as well as on the knowledge they received during their graduate course.

## Data Treatment

The data bank was appraised by Epi Info™ version 3.3.2, and the chi square statistical test was used. Brazilian research shows a minimum age of 15 for the beginning of sexual activity. For this reason, the adolescents studied here were divided into groups of over and under 15 in order to carry out the statistical analysis.

The contraceptive methods were classified according to type, i.e., barrier (male and female condoms, diaphragm, IUD), hormonal (pill, the day-after pill), behavior (billing, abstinence, interrupted sexual relations), and permanent (removal of the uterine tube).

The project was approved by the Federal University of Alagoas (UFAL).

## RESULTS

The sample presented a normal distribution, with average age being 15 and the median and mode 16 (Std Dev 1.8). Most of the group were single (95.7%), did not work (94.1%), lived with both parents (66.3%), and knew of some contraceptive method (95.5%). Of the total, 47% studied at junior schools and 52.4% at high schools (Table 1).

Of the majority of the young women (95.5%) who knew of some contraceptive method, 71% were over 15 years old. The methods mentioned most were the association of hormonal and barrier (72.4%). Abortion and abortive drugs (teas, misoprostol), classified here as "others", were inaccurately indicated as contraceptive methods (Table 1).

Of 559 (21.6%) girls who had had an active sexual life, 32.4% had already been pregnant. Among those, 62.1% were found to be using some kind of contraceptive method, but 37.9% did not use any (Table 2).

In this group, the barrier/hormonal methods were the most well known (75.5%), with the barrier methods being the most used (35.8%). Only four girls (0.7%) used the insecurity behavioral method (interrupted sexual intercourse). There was no significance in the association between age, acquaintance with methods, having an active sexual life, and the kind of method used (Table 2).

As shown in Table 3, in spite of the fact that the majority of the teenagers who had been pregnant before used some kind of contraceptive method (56.4%), many girls (43.6%) did not use any. The majority of teenagers that did not get pregnant used contraceptive methods.

There was no significant association between age, pregnancy, and knowledge of contraceptive methods. There was statistical significance in the use of some kind of contraceptive method and pregnancy (chi square = 343.79; $p < 0.05$). However, in the sexually active group, there was no statistical significance between the use of any contraceptive method and pregnancy (chi square = 0.0322; $p = 0.8576$) (Table 4).

## DISCUSSION

The knowledge and use of contraceptive methods in Brazil is diffuse and has the support of public policies for family planning[21,23]. However, for the girls in our population, this has not been sufficient enough to change their behavior or encourage them to use contraception effectively. Due to behavioral characteristics inherent to their age, the girls are accustomed to run away from educational opportunities related to sexual information and, even when they know the necessary preventive instructions, they do not use them[13,16,20].

In this study, the distribution of adolescents according to their active sexual life and use of contraceptive methods showed that 37.9% of them did not make use of any method, an attitude that leaves them exposed to the risks of an unprotected sexual life (Table 2). This is confirmed by the data, which identified known and used methods, as well as by the citation and use of abortive methods as a way to avoid pregnancy (Tables 1 and 2).

The fact that there were 79 teenagers (43.6%) who had already been pregnant and did not make use of any contraceptive method is also a point to be highlighted in a population that does not want to be pregnant (Table 3).

The results found here have corroborated the specialized literature that points to the youngsters' knowledge of contraceptive methods, in spite of their failure to use them correctly, a reality that exposes them to the risks of sexually transmitted diseases and unplanned pregnancies[6,13,15,18].

There are many reasons that female teenagers get pregnant earlier and earlier, such as age of menarche, precocious sexual initiation, and nonuse of contraceptive methods. In Brazil, and in other developing countries, much attention is given to the socioeconomic reasons that are associated with a precocious sexual initiation and to unknown or minimal use of contraceptive methods[6,7,8,16]. Generally, girls in lower social classes have few plans for the future, such as a profession, and marriage is often a goal of life.

**TABLE 1**
**Characterization of the Research**

| Characteristics | Age | | | | Total | |
|---|---|---|---|---|---|---|
| | 12–15 | | 15–19 | | n = 2,592 | |
| | n = 775 | % | n = 1,817 | % | n | % |
| Level of education | | | | | | |
| Junior school | 751 | 60.6 | 488 | 39.4 | 1,239 | 47.8 |
| High school | 24 | 1.8 | 1,329 | 98.2 | 1,353 | 52.2 |
| Work | | | | | | |
| Yes | 14 | 9.2 | 138 | 90.8 | 152 | 5.9 |
| No | 761 | 31.2 | 1,679 | 68.8 | 2,440 | 94.1 |
| Residence | | | | | | |
| With parents | 550 | 32.0 | 1,168 | 70.0 | 1,718 | 66.3 |
| Mother | 166 | 28.4 | 418 | 71.6 | 584 | 22.5 |
| Father | 9 | 27.3 | 24 | 72.7 | 33 | 1.3 |
| Other relatives* | 48 | 27.3 | 128 | 72.7 | 176 | 6.8 |
| Partners | 2 | 3.0 | 65 | 97.0 | 67 | 2.6 |
| Others** | 0 | 0.0 | 14 | 100.0 | 14 | 0.5 |
| Marital status | | | | | | |
| Single | 762 | 30.7 | 1,719 | 69.3 | 2,481 | 95.7 |
| Married | 13 | 11.7 | 98 | 88.3 | 111 | 4.3 |
| Sexual life | | | | | | |
| Yes | 54 | 9.7 | 505 | 90.3 | 559 | 21.6 |
| No | 721 | 35.5 | 1,312 | 64.5 | 2,033 | 78.4 |
| Know contraceptive methods | | | | | | |
| Yes | 720 | 29.0 | 1,755 | 71.0 | 2,475 | 95.5 |
| No | 55 | 47.0 | 62 | 53.0 | 117 | 4.5 |
| Contraceptive methods known | | | | | | |
| Barrier | 170 | 63.0 | 100 | 37.0 | 270 | 10.4 |
| Hormonal | 20 | 38.5 | 32 | 61.5 | 52 | 2.0 |
| Barrier, hormonal | 466 | 24.8 | 1,411 | 75.2 | 1, 877 | 72.4 |
| Barrier, behavioral | 4 | 50.0 | 4 | 50.0 | 8 | 0.3 |
| Barrier, hormonal, behavioral | 30 | 18.1 | 136 | 81.9 | 166 | 6.4 |
| Barrier, hormonal, behavioral, others | 2 | 40.0 | 3 | 60.0 | 5 | 0.2 |
| Barrier, hormonal, definitive | 3 | 18.7 | 13 | 81.3 | 16 | 0.6 |
| Barrier, hormonal, others | 17 | 23.6 | 55 | 76.4 | 72 | 2.8 |
| Barrier, others | 8 | 88.9 | 1 | 11.1 | 9 | 0.4 |
| None | 55 | 47.0 | 62 | 53.0 | 117 | 4.5 |
| Pregnancy | | | | | | |
| Yes | 13 | 7.2 | 168 | 92.8 | 181 | 7.0 |
| No | 762 | 31,6 | 1,649 | 68.4 | 2,411 | 93.0 |

Notes: *Grandparents, grandmother, aunt, cousin; ** godmother, alone, boss.

**TABLE 2**
**Distribution of the Adolescents with an Active Sexual Life According to Knowledge, Use of Contraceptive Methods, and Pregnancy**

| Distribution | Age | | | | Total | |
|---|---|---|---|---|---|---|
| | 12–15 | | 15–19 | | n = 559 | |
| | n = 54 | % | n = 505 | % | n | % |
| Use of contraceptive methods | | | | | | |
| Yes | 31 | 8.9 | 316 | 91.1 | 347 | 62.1 |
| No | 23 | 10.8 | 189 | 89.2 | 212 | 37.9 |
| Pregnancy | | | | | | |
| Yes | 13 | 7.2 | 168 | 92.8 | 181 | 32.4 |
| No | 41 | 10.8 | 337 | 89.2 | 378 | 67.6 |
| Known contraceptive methods | | | | | | |
| Barrier | 8 | 26.7 | 22 | 73.3 | 30 | 5.3 |
| Hormonal | 1 | 7.7 | 12 | 92.3 | 13 | 2.3 |
| Barrier, hormonal | 34 | 8.1 | 388 | 91.9 | 422 | 75.5 |
| Barrier, hormonal, behavioral | 5 | 9.4 | 48 | 90.6 | 53 | 9.5 |
| Barrier, hormonal, behavioral, others | 0 | 0.0 | 1 | 100.0 | 1 | 0.2 |
| Barrier, hormonal, definitive | 0 | 0.0 | 6 | 100.0 | 6 | 1.1 |
| Barrier, hormonal, others | 3 | 15.8 | 16 | 84.2 | 19 | 3.4 |
| Barrier, others | 1 | 100.0 | 0 | 0.0 | 1 | 0.2 |
| Hormonal, behavioral | 0 | 0.0 | 1 | 100.0 | 1 | 0.2 |
| Others | 0 | 0.0 | 2 | 100.0 | 2 | 0.4 |
| None | 2 | 18.2 | 9 | 81.8 | 11 | 1.9 |
| Used contraceptive methods | | | | | | |
| Barrier | 21 | 10.5 | 179 | 89.5 | 200 | 35.8 |
| Hormonal | 4 | 4.8 | 79 | 95.2 | 83 | 14.8 |
| Barrier, hormonal | 4 | 7.4 | 50 | 92.6 | 54 | 9.7 |
| Behavioral | 2 | 50.0 | 2 | 50.0 | 4 | 0.7 |
| Barrier, hormonal, others | 0 | 0.0 | 3 | 100.0 | 3 | 0.5 |
| Others | 0 | 0.0 | 3 | 100.0 | 3 | 0.5 |
| None | 23 | 10.7 | 189 | 89.3 | 212 | 37.9 |

We found the medium age of 15 to be the age when most teenagers start sexual life and presume that the school, as the place where the data survey took place, is compatible with the Brazilian literature used in the study[24]. As stated above, this precocious age at which adolescents start their sexual lives in Brazil is directly linked to a precarious socioeconomic context, together with the absence of the participation of both the school and health service departments in the process of adolescent sexual education[24,27].

In this study, it was also observed that teenagers who learned how to use contraceptive methods, 347 (62.1%) (Table 2), and adequately use them were less likely to have become pregnant than those who had been pregnant before 79 (11.3%) and yet do not use any method (Table 3), a condition that exposes them to the risks of a new pregnancy. This fact was proven by the presence of a significant association between the use of contraceptive methods and the girls that had never been pregnant before (Table 4).

**TABLE 3**
**Distribution of the Adolescents According to Use of Contraceptive Methods and Pregnancy**

| Distribution | Age | | | | Total | |
|---|---|---|---|---|---|---|
| | 12–15 | | 15–19 | | | |
| | n = 54 | % | n = 505 | % | n = 559 | % |
| Got pregnant | 13 | 7.2 | 168 | 92.8 | 181 | 32.4 |
| Used some contraceptive method | 6 | 5.9 | 96 | 94.1 | 102 | 56.4 |
| Did not use any contraceptive method | 7 | 8.9 | 72 | 91.1 | 79 | 43.6 |
| Not pregnant | 41 | 10.8 | 337 | 89.2 | 378 | 67.6 |
| Do not use any contraceptive method | 16 | 10.2 | 117 | 89.8 | 133 | 35.2 |
| Use some contraceptive method | 25 | 12.0 | 220 | 82.0 | 245 | 64.8 |

**TABLE 4**
**Chi Square Result**

| Variable Independent | | Variable Dependent | Statistical Test | Result |
|---|---|---|---|---|
| Use contraceptive method | All teenagers | Pregnant | Chi square = 343.79 $p < 0.05$ | S |
| Use contraceptive method | Teenagers with active sexual life | Pregnant | Chi square = 0.73 $p > 0.05$ | NS |

S, significant; NS, nonsignificant.

The reason that barrier methods are the most cited and used in this study reflects the encouragement of the use of condoms for the prevention of AIDS[6,7,8,9,10,11,14] at the national and international level (Tables 1 and 2)

The hormonal methods, also frequently mentioned here (Table 2) and mostly represented by monthly birth control pills, strengthen the references that were studied that demonstrate how important these pills are for a woman's sexual life, i.e., a life free of risks associated with an unplanned pregnancy[12,21].

Presently, the less developed countries are those that present more contraception-related problems and, consequently, the greatest rates of fertilization and birth in adolescents[6,19,20,21,22].

This study shows that contraceptive methods are still unknown for 4.5% of the girls who participated in the study, as well as the 37.9% with active sexual lives who do not use them (Tables 1 and 2). Brazil has promoted public campaigns to inform youngsters about the use of contraceptives[11,21,24]; however, the results have been unsatisfactory as shown by the increase in the incidence of AIDS and birth rates at this age level[23,26,27,28,29].

The erroneous citation of abortion as a contraceptive method, together with the use of medications like misoprostol, calls attention to the need to discourage its utilization by young Brazilian girls to resolve their problems of an unplanned pregnancy. It does, however, confirm the literature used in this study, which points to the use of a drug such as misoprostol as a way for young Brazilian girls to achieve an abortion, even though the practice is illegal and the indiscriminate use of this medicine is prohibited[10,15,20,26].

## CONCLUSION

The girls who participated in this study know contraceptive methods, even though many of those who are over 15 still expose themselves to the risks by not using them. The barrier methods were the most mentioned and used. By taking into consideration the inadequate use of condoms and the small use of hormonal methods, the study shows that there is an exposure to the risks of pregnancy and illness, an issue that will be addressed in future studies. It also emphasizes the incorrect citation of abortion and abortive drugs as contraceptive methods, which can stimulate the nonuse of pregnancy preventive measures.

## ACKNOWLEDGMENTS

This project was financed by the Foundation of Support to the Research in the State of Alagoas (FAPEAL).
